# Harnessing the power of Raman spectroscopic imaging for ophthalmology

**DOI:** 10.3389/fchem.2023.1211121

**Published:** 2023-05-12

**Authors:** Jing Li, Peirao Yan, Yong Li, Ming Han, Qi Zeng, Juan Li, Zhe Yu, Dongjie Zhang, Xueli Chen

**Affiliations:** ^1^ Shaanxi Eye Hospital, Xi’an People’s Hospital (Xi’an Fourth Hospital), Affiliated People’s Hospital of Northwest University, Xi’an, Shaanxi, China; ^2^ Center for Biomedical-photonics and Molecular Imaging, Xi’an Key Laboratory of Intelligent Sensing and Regulation of Trans-Scale Life Information, School of Life Science and Technology, Xidian University, Xi’an, Shaanxi, China

**Keywords:** Raman spectroscopic imaging, ophthalmology, retina, lens, multimodality imaging

## Abstract

Eye diseases can cause great inconvenience to people’s daily life; therefore, it is necessary to study the causes of ocular diseases and related physiological processes. Raman spectroscopic imaging (RSI) is a non-destructive, non-contact detection technique with the advantages of label-free, non-invasive and highly specific. Compared with other mature imaging technologies, RSI can provide real-time molecular information and high-resolution imaging at relatively low cost, making it very suitable for quantitative detection of biological molecules. RSI can reflect the overall situation of the sample, revealing the content distribution of the same substance in different areas of the sample. This review focuses on the recent advances in ophthalmology, with particular emphasis on the powerful use of RSI techniques, as well as its combination with other imaging techniques. Finally, we prospect the wider application and future potential of RSI approaches in ophthalmology.

## Introduction

Ophthalmology is a specialty that studies diseases related to the visual system and focuses on a variety of ophthalmic diseases, including retinal diseases, glaucoma, optic neuropathy, and cataracts (Santos et al., 2020; Thompson et al., 2021; Cicinelli et al., 2023; Miao et al., 2023). The occurrence of eye diseases may correspond to some changes in the internal structure and chemical components of the eyes. Detection of the corresponding sites facilitates rapid and accurate diagnosis, screening, and classification of the diseases (Sheerin et al., 2012; Géhl et al., 2016; Matlach et al., 2017; Schmid et al., 2021; Sullivan et al., 2021). At present, the commonly used examination methods for ophthalmology in clinical practice are mostly to evaluate the changes in eye structure and color, such as fundus photography, fundus B ultrasonography, and optical coherence tomography (OCT) (Lü and Qin, 2020; Propst et al., 2020; Wong et al., 2020). The quantitative information of biochemical components in eye tissue can only be obtained by invasive techniques, such as biopsy or needle absorption, and *in vitro* detection (Schefler et al., 2013; Wong et al., 2021).

However, Raman spectroscopy does not cause any damage to the samples, providing a non-invasive way for the rapid diagnosis of ocular diseases. Raman spectroscopy can display abundant structural information about molecules, and the required sample volume is small (Das and Agrawal, 2011). In addition, since the Raman signal of water is relatively weak compared with the infrared technology, the Raman spectroscopy technology is more suitable for the detection of biological molecules and is of great significance for the detection of clinical samples (Shipp et al., 2017). Raman spectroscopic imaging (RSI) technology presents the information of multiple sample points at the same Raman peak on a pseudo-color image, which can directly observe the distribution of biochemical components in the pathological structure and help to study the causes of diseases (Shen et al., 2019). There are many studies that apply RSI to the related fields of ophthalmic diseases, including spontaneous Raman scattering, surface-enhanced Raman scattering (SERS), stimulated Raman scattering (SRS), and coherent anti-Stokes Raman scattering (CARS) imaging.

In this review, we focus on the contribution of Raman spectroscopic imaging as a research tool in the field of ophthalmology. The powerful use of RSI techniques combined with other imaging techniques is also concluded. Finally, we prospect the wider application and future potential of RSI approaches in ophthalmology.

## RSI for ophthalmic applications

Currently, RSI has many applications in the field of biomedicine. Specifically for application research in the field of ophthalmology, Raman spectroscopic imaging technology focuses more on ocular structures such as the retina, lens, cornea, and vitreous humor.

### The retina

The retina, also known as peripheral brain, is an extension of brain tissue that is directly connected with the outside. Therefore, the changes of biomolecular content in retina can not only reflect the pathological changes of the retina (such as glaucoma and macular degeneration), but also help us understand the health conditions of brain cells and other parts of the body (such as diabetes and Alzheimer’s disease) (Gellermann et al., 2002b; Hoon et al., 2014; Lee et al., 2022).

**FIGURE 1:**
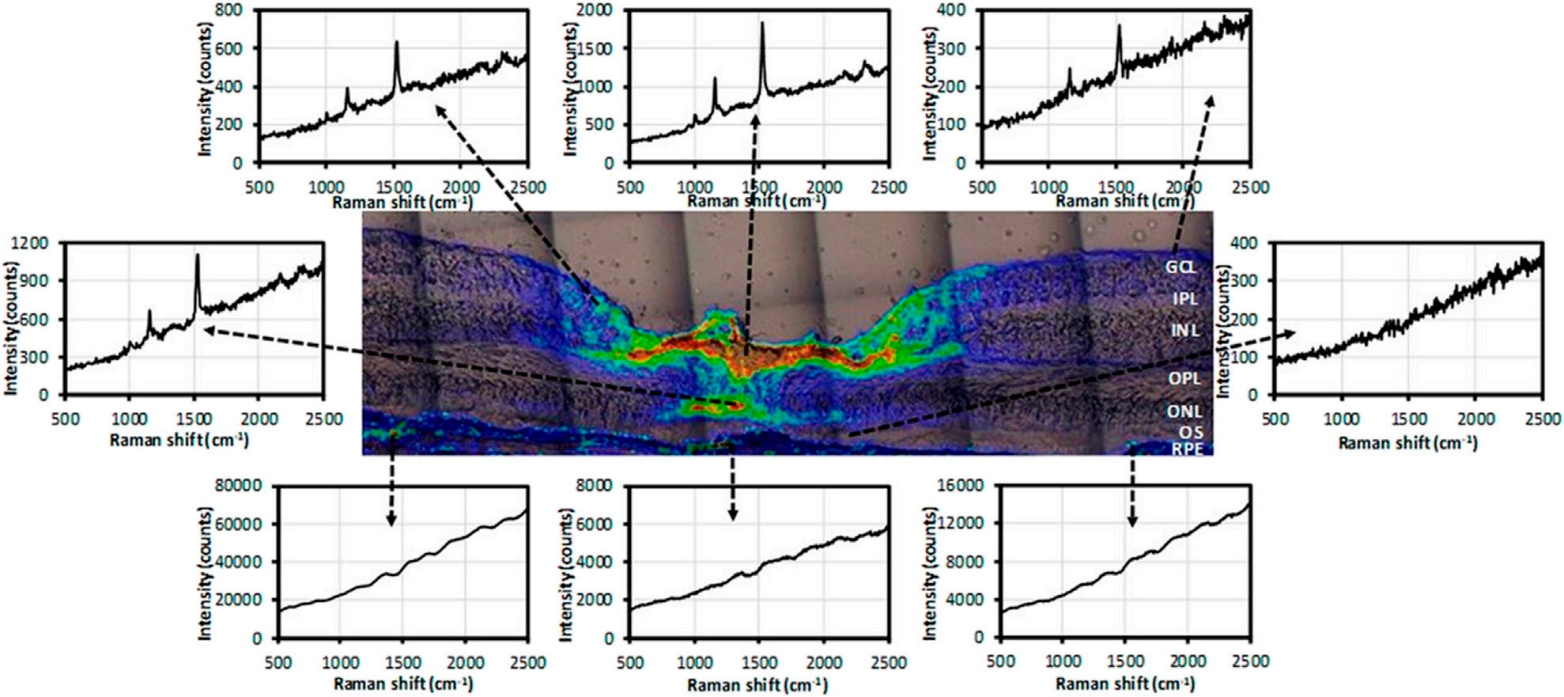
Raman spectra at selected locations in the intensity map of total carotenoids (Li et al., 2020).

Lutein and zeaxanthin are carotenoids concentrated in the macula of the retina and have the effect of preventing oxidative damage to the eye, but they differ only by a C=C bond position, which cannot be accurately distinguished by the existing technology (Gellermann et al., 2002a; Ermakov et al., 2005). Li et al. imaged lutein and zeaxanthin in the human retina based on confocal resonance Raman microscopy. As shown in Figure 1, most of the zeaxanthin was concentrated in the fovea, while lutein was distributed in the macula at a relatively low concentration. And it was considered that zeaxanthin might play a more important role in human macular health and disease than lutein. Retinal dopamine is considered to be related to the development of myopia (Li et al., 2020). Ren et al. used SERS to image dopamine in the retina of form-deprivation (FD) mice before and after treatment, realizing high sensitivity and high specificity for detection in the range as low as the nanomolar range. This method is helpful to evaluate the therapeutic effect of myopia (Ren et al., 2021). Stiebing et al. performed *in vitro* Raman spectroscopic imaging detection on mice retina, observed the retinal layered structure, and distinguished the healthy and Alzheimer’s disease retinal tissues according to the related Raman information with accuracy of 85.9% (Stiebing et al., 2020). Notably, Sharifzadeh et al. realized Raman spectroscopic imaging of the distribution of macular pigment in the retina *in vivo* for the first time, found that macular pigment is usually present in relatively high concentrations in healthy human macula relative to the peripheral retina (Sharifzadeh et al., 2008).

In summary, Raman spectroscopic imaging can quickly and non-destructively analyze isolated and even *in vivo* retina, which can help complete the early diagnosis of related diseases and evaluate the effectiveness of treatment (Li et al., 2020).

### Lens

In the human lens, protein accounts for 60% of its wet weight and the vast majority of its dry weight (Roskamp et al., 2020). The orderly arrangement of lens proteins ensures the transparency and refractive gradient of the lens. Ageing or diseases in other parts of the body may cause degeneration of lens proteins, which may lead to lens presbyopia or cataracts (Wishart et al., 2021). Therefore, the conformation of proteins in the lens can be examined with the help of RSI to analyze the cause of the disease and its physiological processes (Uzunbajakava et al., 2003; Sacharz et al., 2016).

**FIGURE 2:**
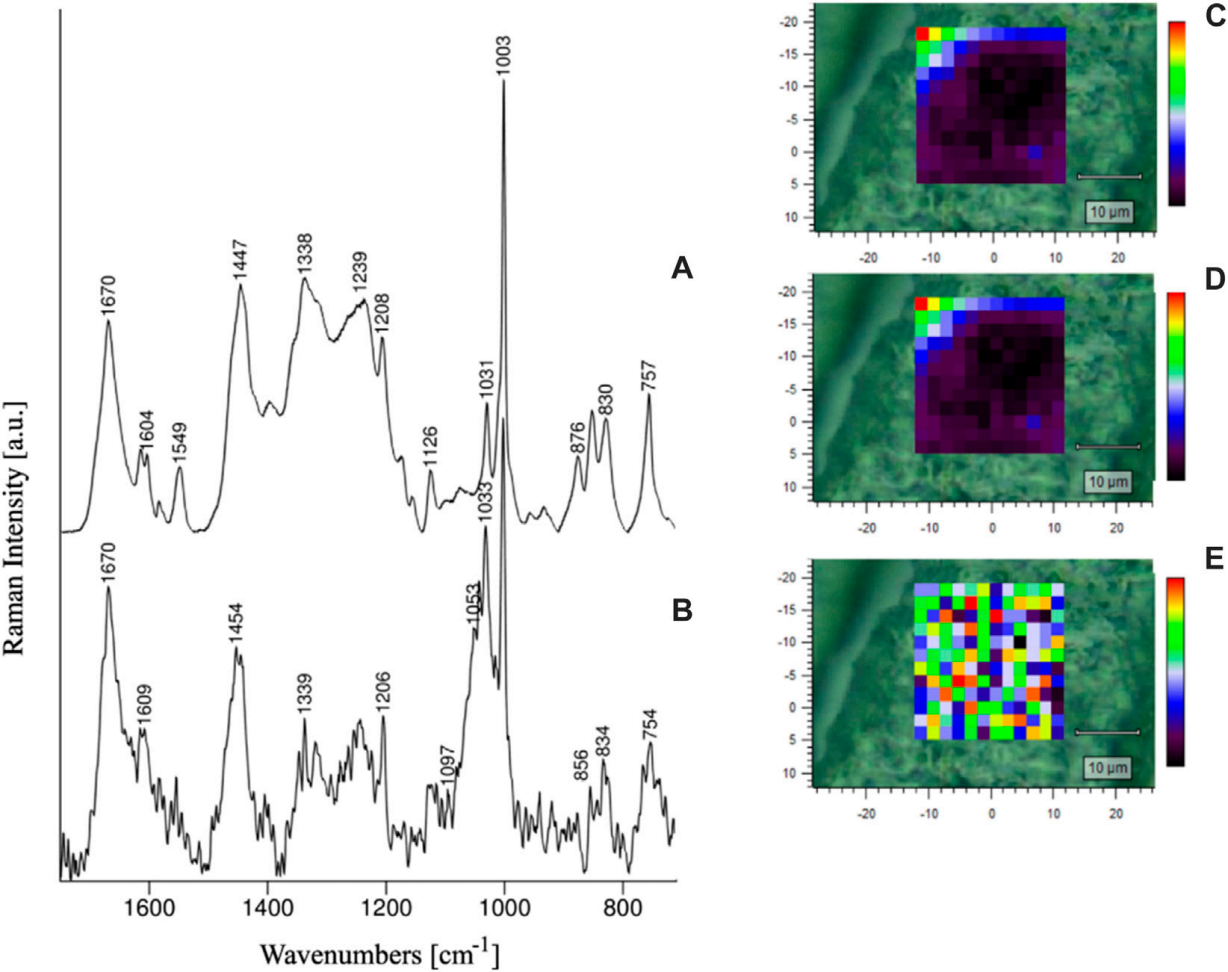
RS spectra of healthy human lens **(A)** and cataractous human lens **(B)**, as well as Amide I/1670 cm^-1^ **(C)**, Amide II/1549 cm^−1^ **(D)** and Amide I/Amide II ratio intensity maps **(E)** of the healthy lens (Paluszkiewicz et al., 2018).

Paluszkiewicz et al. analyzed the conformational changes of amino acid residues in the lens of healthy individuals and cataract patients using Raman spectroscopy and RSI. The results showed that the lens samples from healthy humans and cataract patients differed in the conformation of Tyr and Trp residues and protein secondary structure (Figure 2) and that the development of cataract disease did not occur uniformly throughout the volume of the lens (Paluszkiewicz et al., 2018). Michael et al. applied RSI to the study of Alzheimer’s disease. Comparison of the protein spectrum of plaques and tangles in the hippocampus of the deceased patient’s brain and the protein spectrum of the lens by RSI revealed a high concentration of amyloid-β in the hippocampus of the brain and a relatively low level of amyloid-β in the lens. Therefore, cortical cataract and Alzheimer’s disease may not be causally related, and cortical cataract cannot be considered an indicator or predictor of Alzheimer’s disease (Michael et al., 2014).

### Other eye structures

Currently, some studies have evaluated the distribution of lipids in different ocular structures (such as the cornea and meibomian gland) by RSI and analyzed the related physiological processes through the accumulation of lipids in different regions (Segawa et al., 2015; Jester et al., 2016).

Ammar et al. used vibrational oscillations of carbon-hydrogen bonds in lipid membranes to achieve label-free, high-resolution CARS mapping of cells and non-cellular structures in the intact mouse cornea and corneal limbus (Ammar et al., 2013). Paugh et al. explored the difference in the composition of palpebral lipid and protein detected by hyperspectral stimulated Raman scattering (hsSRS) microscopy in patients with meibomian gland dysfunction, including normal subjects and patients with evaporative dry eye. The lipid-protein component was found to be associated with tear film rupture time (TBUT), suggesting that hsSRS can be used for meibomian gland quality classification and therapeutic evaluation (Paugh et al., 2019). Kim et al. studied the ability of eicosapentaenoic acid (EPA) to activate the PPAR γ signal pathway through SRS. The results showed that EPA induced meiocyte differentiation of meibomian through PPAR γ activation. What’s more, they have imaged the whole process of lipid synthesis and extracellular transport to form lipid droplets located in the endoplasmic reticulum (Kim et al., 2020).

In summary, RSI has been successfully applied to several ocular structures to detect the content and distribution of various biochemical components in them, which can visually demonstrate the difference between normal individuals and patients from a biochemical perspective and facilitate the monitoring of physiological processes in ocular structures, making it a powerful tool for the study of ocular biochemistry.

## RSI combining with other technologies for ophthalmic applications

RSI can be used in conjunction with other imaging techniques to obtain multivariate information about the sample, in addition to being applied individually to the ophthalmic field. For example, optical coherence tomography (OCT), two-photon autofluorescence imaging (TPAF), two-photon excitation fluorescence (TPEF), and second harmonic generation (SHG) (Evans et al., 2009; Masihzadeh et al., 2013; He et al., 2015; Kaji et al., 2017). There are many technical challenges in combining these imaging methods with RSI. At present, researchers have developed a few detection systems that can simultaneously realize the combination of Raman spectroscopic imaging with other imaging technologies such as OCT and TPAF, and have been successfully applied to ophthalmology.

OCT is currently commonly used clinically as an imaging device for the diagnosis and monitoring of the retina and macula, helping to improve people’s understanding of the structure of the eye. Evans et al. developed a system combining OCT and Raman spectroscopic imaging to simultaneously obtain chemical and structural information of Cytochrome c and proved the possibility of application of the system to retinal imaging *in vivo* (Evans et al., 2009).

TPAF and CARS are both multiphoton microscopy techniques that which have been used to image the retinas of living nonhuman primates. However, due to the low fluorescence of the cell, the detection time is generally long. Combining TPAF and CARS imaging technology, we can obtain the fluorescence signal and Raman signal of tissue at the same time, and there is no need for fluorescent labeling proteins (Ammar et al., 2013). Masihzadeh et al. designed a dual-mode imaging system of TPAF and CARS and imaged the newly excised mice eyes. The results showed that the fluorescence signal of collagen fibers in the sclera was obvious in the TPAF channel, while the rod-shaped outer segment could be identified by the CARS signal of the lipid-rich plasma membrane, which was helpful to further understand retinal function and dysfunction (Masihzadeh et al., 2013).

## Discussion and prospects

So far, RSI has been successfully applied to the detection of various eye structures such as the retina, lens, and cornea, and has achieved the collection of Raman spectroscopic images of biochemical components such as macular pigment, dopamine, and protein in different parts. Therefore, the application of Raman spectroscopic images in ophthalmology will help us understand the local distribution of certain disease marker molecules before and after disease occurrence or treatment, as well as the study of disease physiological processes. At the same time, the combination of RSI and other imaging technologies can simultaneously obtain multivariate information of samples.

Numerous studies have demonstrated the potential value of Raman spectroscopic imaging in the diagnosis of ophthalmic diseases. However, there are still several difficulties in the application of RSI in ophthalmology. Firstly, most studies are in the *in vitro* experimental stage, and only Sharifzadeh et al. achieved *in vivo* Raman spectroscopic imaging detection of the human retina in 2008 (Sharifzadeh et al., 2008). Higher requirements have been put forward for Raman spectroscopic imaging systems in physical examinations, such as the convenience of detection and laser safety. Secondly, it is also a challenge to accurately match the characteristic information of Raman spectroscopy with the physiological and pathological characteristics of ophthalmic diseases. Finally, the accuracy of analyzing and diagnosing eye diseases using Raman spectroscopic imaging needs to be improved. Based on the problems faced by the application of Raman spectroscopic imaging in ophthalmic research mentioned above, we look forward to further research and breakthroughs in the following three areas: firstly, at the technical level, in order to achieve *in vivo* detection, the instrument can be miniaturized and Bessel beams can be introduced as excitation light sources. On the one hand, Bessel beams can reduce light illumination, and on the other hand, they help to obtain better spectra in scattering media (Wang et al., 2022). At the same time, within the excitation power range allowed by international laser safety regulations, non-resonant Raman spectroscopy can be combined. Stiebing et al. have demonstrated the feasibility of *in vitro* detection of human retina using non-resonant Raman spectroscopy in 2019 (Stiebing et al., 2019). Secondly, at the application level, SERS molecular probe technology can be combined with Raman spectroscopic imaging technology to achieve specific detection of eye structures and improve detection sensitivity. Thirdly, at the methodological level, Raman spectroscopic imaging can be combined with artificial intelligence technology, utilizing artificial intelligence and deep learning technology to assist in the intelligent analysis of ophthalmic Raman spectral data for disease diagnosis. With the development of technology, Raman spectroscopic imaging is expected to achieve clinical diagnosis of ophthalmic diseases, which is of great significance for early diagnosis and pathological research of ophthalmic diseases.
